# Mental health problems raise the odds of cognitive impairment in COVID-19 survivors

**DOI:** 10.3389/fpsyt.2024.1370085

**Published:** 2024-08-14

**Authors:** Madhushree Chakrabarty, Piali Chatterjee, Adreesh Mukherjee, Gautam Das, Rafikul Islam Mollah, Banshidhar Mondal, Swarup Sardar, Ayanendranath Basu, Mrinalkanti Ghosh, Amitabha Sengupta, Sankar K. Pal, Atanu Biswas

**Affiliations:** ^1^ Department of Neuromedicine, Bangur Institute of Neurosciences, Kolkata, India; ^2^ Department of Neuromedicine, Institute of Post Graduate Medical Education & Research and Seth Sukhlal Karnani Memorial (SSKM) Hospital, Kolkata, India; ^3^ Department of Neuromedicine, North Bengal Medical College, Siliguri, India; ^4^ Interdisciplinary Statistical Research Unit (ISRU), Indian Statistical Institute, Kolkata, India; ^5^ Department of Radiology, Burdwan Medical College, Bardhaman, India; ^6^ Center for Soft Computing Research, A National Facility (CSCR), Indian Statistical Institute, Kolkata, India

**Keywords:** COVID-19, cognition, anxiety, depression, stress, insomnia, long-term-effects, mental health

## Abstract

**Background:**

COVID-19 survivors around the globe are suffering from mental health issues. While mental health problems can be an early warning sign of dementia, they may also increase the chances of developing the disease. In this study, we examined the mental health of COVID-19 survivors and mapped its associations with cognitive and demographic variables.

**Method:**

COVID-19 survivors listed in the databases of three tertiary care hospitals in Kolkata were contacted sequentially. 376 willing patients were interviewed over the telephone. 99 COVID-19 patients and 31 matched controls participated in the in-person interviews that were arranged for a more detailed investigation. The participants were administered standardized tests that are widely used for the assessment of cognitive functioning and mental health status.

**Result:**

64.89% of COVID-19 survivors reported a deterioration in physical functioning. 44.95% reported a decline in mental health, whereas 41.49% reported a drop in cognitive performance. Detailed investigations revealed that they had an increased risk of having depression, anxiety, and poor sleep quality by 91%, 68%, and 140%, respectively. 6.1% of the patients had mild cognitive impairment, and 4% had dementia. COVID-19 patients who had depression and anxiety were 8.6 and 19.4 times more likely to have cognitive decline, respectively. Compared to the matched controls, COVID-19 patients had greater depression (p<.001), anxiety (p<.001), stress (p =.003), and insomnia (p <.001). They also scored significantly lower on Addenbrooke’s Cognitive Examination-III (p =.009) and Picture Naming Test (p =.005) and took significantly longer to complete Trail Making Test-A (p =.002).

**Conclusion:**

COVID-19 survivors in this study had major mental health issues even one year after contracting the virus. They had significant cognitive deficits that might progress into dementia. Strict monitoring and systematic treatment plans should be implemented as soon as possible.

## Introduction

COVID-19 has caused significant distress around the globe. According to a report released by the World Health Organization (WHO), the global prevalence of anxiety and depression has escalated by a massive 25% (1). In India, the situation is far more somber. A study found that 40.5% of 1,685 participants were suffering from typical mental health illnesses like anxiety or depression, and 71% had poor well-being due to the pandemic (2).

COVID-19 survivors around the world have been reported to suffer from mental health issues (3–9). Maley et al. reported that 80% of the COVID-19 survivors who required mechanical ventilation support had post-intensive-care syndrome (4). 33% had significant symptoms of post-traumatic stress, 38% had anxiety, and 42% had depression (4). Nakamura et al. (10) stated that more than 30% of patients hospitalized with COVID-19 might have cognitive impairment, depression, and anxiety long after their discharge. The study suggested that patients who required treatment at the intensive care unit for severe COVID-19 infections were at greater risk for the development of these symptoms (10).

Coronavirus has frequently been linked to damage to the central nervous system (11, 12). Studies have shown that the severe acute respiratory syndrome coronavirus (SARS-CoV) and the Middle East respiratory syndrome coronavirus (MERS-CoV) affect brain areas like the cortex, subcortical areas, hypothalamus, and white matter (13, 14). Recent studies claim that having a COVID-19 infection raises the likelihood of developing dementia (15–23). While mental health problems can be an early indicator of dementia, chronic mental health issues might increase the risk of developing dementia (24–28). Mental health is intricately interrelated with neurological functioning and, consequently, with cognitive functioning. For example, pathological anxiety and chronic stress might cause structural degeneration and decreased functioning of the hippocampus and prefrontal cortex, thereby increasing the risk of developing neuropsychiatric illnesses such as depression and dementia (29). Similarly, cortical brain areas implicated in depression (dorsal and medial prefrontal cortex, dorsal and ventral anterior cingulate cortex, the orbital frontal cortex, and the insula) (30) are also associated with numerous cognitive functions such as consciousness, attention, salience processing, emotion processing, social cognition, taste perception, hedonic and aversive responses to odors, self-awareness, decision-making, etc. (31–35). Hence, mental health problems can lead to cognitive impairment.

Lingering mental health problems long after recovery from COVID-19 infection have been reported in numerous studies (36). However, there is a paucity of empirical research on cognitive impairments associated with long-term psychiatric problems in COVID-19 survivors beyond one year of contracting the infection. The few studies that have investigated this aspect suffer from methodological limitations. The use of different tools and methods reduces the interpretability of the results (37, 38).

Moreover, the majority of the studies focusing on the mental health of COVID-19 survivors are from China (6, 38–41). Although mental health problems occur in all cultures and groups, the stage, severity, and form of the presenting symptoms might differ greatly (42). Cross-cultural evidence is imperative for gaining a deeper insight into the intricacies associated with neuropsychiatric impairment in COVID-19 patients. India has the greatest burden of mental and behavioral disorders, which, according to the WHO, is approximately 2443 disability-adjusted life years (DALYs) per 100,000 population (43). India also has a high prevalence of dementia (7.4%) (44). However, we do not have any studies on the Indian population, investigating the impact of psychiatric illness on the cognitive health of COVID-19 survivors.

In this study, we investigated depression, anxiety, perceived stress, and insomnia in COVID-19 survivors, as well as their correlations with different cognitive tests, clinical parameters, and demographic factors. Widely used tests were administered in offline testing sessions after initial telephone interviews.

## Methods

### Settings

Data were collected from July 27^th^, 2022, to November 30^th^, 2023. Telephonic interviews were conducted over the phone based on a structured questionnaire. Face-to-face interviews and clinical examinations were done either in a quiet room at the Bangur Institute of Neurosciences, Kolkata, or at the participant’s home by trained personnel. Verbal consents were obtained for the telephonic interviews after having read a scripted version of the consent form with detailed information about the study. All in-person interview participants signed an informed consent form approved by the institutional ethics committee (Memo No.: IPGME&R/IEC/2021/639).

### Sample size

The dearth of robust studies on neuropsychiatric symptoms in COVID-19 patients in the Indian population posed a problem in sample size estimation. Nalleballe et al. (45) reported that 9086 of 40,469 patients (22.5%) had neuropsychiatric manifestations. We assumed that the proportion observed by Nalleballe et al. (45) (22.5%) was a reasonable estimate of the true proportion and calculated the sample size using this data. The sample size was estimated to be 268, with a precision of 5% and a confidence interval of 95%. We expected at least 20% (20% of 268 = 53.6) of the participants to drop out of the study. So, the required sample size was estimated to be 268 + 53.6 = 322.

However, we could maintain an adequate sample size only for the telephonic interview. Due to a general unwillingness among patients and controls for in-person interviews, we could not meet the required sample size and were compelled to adopt the non-probabilistic sequential sampling technique. We kept scheduling patients and controls who were willing to participate in the in-person interviews and conducted statistical analysis at regular intervals until we were confident about our results.

We also could not recruit an appropriate number of control subjects. Apart from Asians’ general reluctance to participate in health research (46–49), the fact that Kolkata was one of the hardest-hit regions, with a large number of infected people, could have contributed to the difficulties in obtaining non-infected controls in our study (50).

### Participants

COVID-19 survivors from three tertiary care hospitals in Kolkata were sequentially contacted over the phone. 392 patients responded with enthusiasm and agreed to complete a brief questionnaire on their COVID-19 symptoms, therapy, and current health status. Sixty-eight of them did not complete the interview. They were probably apprehensive about sharing their personal details and health information due to security and privacy concerns. Asians worldwide are less likely than other racial groups to participate in health research (46–48), which could explain why it is difficult to find willing participants. This disinclination could be attributed to their lack of trust in society in general and the health system in particular, especially after the pandemic (49, 51).

Contrary to the reluctance of the individuals mentioned above, a few requested that their non-hospitalized family members be included, so we interviewed 52 non-hospitalized COVID-19 patients with positive RT-PCR test reports. There appears to be no specific rationale for the inclination to include their family members in the research protocol other than greater awareness and concern, particularly for elderly and vulnerable family members.

This study investigated 376 telephone interviews with COVID-19 patients in total (average age: 52.97; SD: 15.37; average education: 13.88; SD: 4.3; male: 227; female: 149; hospitalized: 317; non-hospitalized: 52; urban: 318; suburban: 27; rural: 31; 49.5% of patients required oxygen therapy; 60.9% lost their sense of smell and 60.1% lost their sense of taste; 75.3% were unvaccinated during the COVID-19 infection).

Patients who met the inclusion criteria (adult, right-handed, native Bengali speakers with at least 10 years of formal education and no prior history of neurological or psychological disorders) and agreed to participate in further investigations involving a face-to-face interview and clinical examinations were recruited for a more detailed probe. Non-hospitalized family members of the patients were also invited to take part in the study and were recruited if they fulfilled the inclusion criteria and provided consent to participate in the research.

Control participants were selected based on their self-reported lack of COVID-19 infection and a questionnaire designed to exclude cases that might have had COVID-19 infections despite not having any confirmed test reports. Scores on the General Health Questionnaire (GHQ) were used to exclude patients with significant mental health issues. Four control participants had a GHQ score >15 as well as COVID-19 symptoms such as fever, cold, cough, loss of taste, and smell. They were excluded from the study. One non-infected control participant who scored below the cutoff in Addenbrooke’s Cognitive Examination-III was included in the risk ratio and odds ratio calculations but was excluded from further analyses. The excluded control participants were offered medical support and counseling.

Data from 99 COVID-19 patients (male: 56, female: 43; hospitalized: 71, non-hospitalized: 28) and 31 non-infected controls (male: 18, female: 13) were included in the final analyses. The two groups were comparable in age (p =.103), education (p =.828), socioeconomic status (p =.482), and gender (p =.884) (**Table 1**).

**Table 1:** Comparative analysis of demographic variables between COVID-19 patients and non-infected controls.

	Group	N	Mean	SD	t- Value	df	p-value
Education	Patients	99	15.4	3.1	0.218	128	0.828
	Controls	31	15.3	4.4			
Gender	Patients	99	0.4	0.5	0.146	128	0.884
	Controls	31	0.4	0.5			
Age	Patients	99	52.8	13.9	1.644	128	0.103
	Controls	31	48.0	15.9			
Socioeconomic status	Patients	99	18.9	6.5	-0.705	128	0.482
	Controls	31	19.8	6.5			

### Materials

A structured questionnaire was used for the telephone interviews. The questionnaire was divided into five sections and took approximately 15–20 minutes to complete. The first section of the questionnaire inquired about the participants’ demographics and socioeconomic status. The second contained questions pertaining to participant’s general health, including whether they had a neurological or psychiatric illness before the COVID-19 infection, their vaccination status when they contracted the disease, and any pre- and post-COVID-19 co-morbidities. The third section comprised questions to determine the severity of the COVID-19 infection and inquired about the type of treatment received and its duration. The fourth section comprised questions about the participant’s symptoms during the COVID-19 infection and whether they were still present. The symptoms were categorized into physical, mental, and cognitive. In the fifth section, participants were asked to rate their physical, mental, and cognitive functioning before and after the COVID-19 infection on a scale of 0 to 10. Before rating, participants were explained what physical, mental, and cognitive performance meant.

A more elaborate demographic and clinical data form, specifically tailored for this study, was used to record demographic details and the clinical histories of the control and patients who participated in the in-person interviews. The control participants were also asked to rate their perceived physical, mental, and cognitive functioning before and after COVID-19 on a scale of 0 to 10 (with 0 representing the worst and 10 representing the best).

Additional tests were administered in the in-person interviews that were scheduled after the telephonic interviews. The Addenbrooke’s Cognitive Examination-III (ACE-III) (52) was used to assess overall cognitive functioning, including attention, orientation, memory, language, verbal fluency, and visuospatial skills. Trail-making tests (TMTs) A and B (53) were used to assess visual attention and task switching. It also provided information about visual search speed, scanning speed, processing speed, mental flexibility, and executive functioning. The Digit Span Test (DST) (54) was administered to assess short-term verbal memory. The forward-span task evaluated verbal working memory and attention, and the backward-span task assessed working memory as well as cognitive control and executive functioning. The Picture Naming Test (PNT) (53) was used to assess lexical retrieval. The Verbal Learning Test (VLT) (55) was used to assess recall, an important aspect of verbal memory. The Perceived Stress Scale (PSS) (56), Beck’s Depression Inventory (BDI) (57), Beck’s Anxiety Inventory (BAI) (58), and the Insomnia Severity Index (ISI) were used to measure the mental health of the COVID-19 patients. The WHO clinical progression scale was used to measure the severity of illness (59) in patients. The scale provides a measure of disease severity, ranging from 0 (not infected) to 10 (dead). Modified Kuppuswamy’s Socioeconomic Scale (60) was used to determine the socioeconomic status (SES) of the participants, and the Edinburgh Handedness Inventory (61) was administered to assess their handedness.

### Procedure

All the tests were administered and scored as per the instructions given in the respective test manuals. The second, fifth, sixth, and seventh authors collected and entered the data. They were recruited per the Indian Council of Medical Research’s recommendations for selecting project technical assistants in grades II and I. A qualified clinical psychologist and her team trained them to conduct the neuropsychological and mental health tests.

The percentage of perceived changes in physical, mental, and cognitive functioning was calculated as *(pre-COVID-19 score − post-COVID-19 score) / the patient’s highest score for that health parameter.*

### Statistical analyses

The anonymized data were transferred to SPSS version 21 (IBM SPSS Statistics 21) for statistical analysis.

To get an initial estimate of the impact of the disease on this population, we examined the telephone interviews. The percentage of patients who reported perceived impairments in their physical, mental, and cognitive functioning was assessed. We also calculated Pearson’s correlation coefficients to examine if there were any linear relationships between the demographic variables (age, education), clinical parameters (days in hospital, severity score, number of vaccine doses), and perceived changes in physical, mental, and cognitive changes.

The in-person interviews and standardized test battery scores were also analyzed. The relative risk (RR) and the odds ratio (OR), standard errors, and 95% confidence intervals were calculated following the standard procedure (62–64). For calculating the relative risk and odds ratio of poor performance in ACE-III co-occurring with depression, anxiety, stress, and insomnia, the poor performance was considered to be 1 SD below the mean score obtained by the control group (95.3-2.6 = 92.7 ≈ 93).

To see if there were any significant differences between the two groups, we performed an F-test based on two-sample Hotelling’s T^2^ statistics (65), with the mental health test scores (PSS, BDI, BAI, and ISI) representing the data vectors and the two groups under study being the COVID-19 patients and the non-infected participants.

For the cognitive tests, the dependent variables had very low correlations, so the formation of linear composites was not possible (66). Hence, we conducted independent sample t-tests with each of the cognitive tests (ACE-III, VLT-delayed recall, DST-forward, DST-backward, PNT, and TMT-A and TMT-B) to find out if there were any significant differences between the two groups (COVID-19 patients and non-infected controls). Independent sample t-tests were also done with the five domains of ACE-III (attention, memory, language, fluency, and visuospatial skills) as dependent variables to find out the domains in which the patients and the controls differed.

The Pearson correlation coefficient, also known as Pearson’s *r*, was used to explore if there existed any linear relationships between different mental and cognitive health parameters. We also explored if the number of days spent in the hospital and disease severity had any significant correlations with the scores on mental and cognitive health tests.

Stepwise linear regressions were carried out to find if the demographic variables (age, gender, education, and SES) could predict the mental and cognitive health of COVID-19 patients.

## Result

### Result of the telephone interviews

#### Analysis of the perceived health status

Patients reported a decline in their physical (average decline: 30.35%, SD: 16.46), mental (average decline: 32.04%, SD: 9.04), and cognitive functioning (average decline: 29.11%, SD: 17.88). 10.6%, 9%, and 6.9% of patients, respectively, reported at least a fifty percent drop in physical, mental, and cognitive performance.

#### Correlations

**Figure 1:**
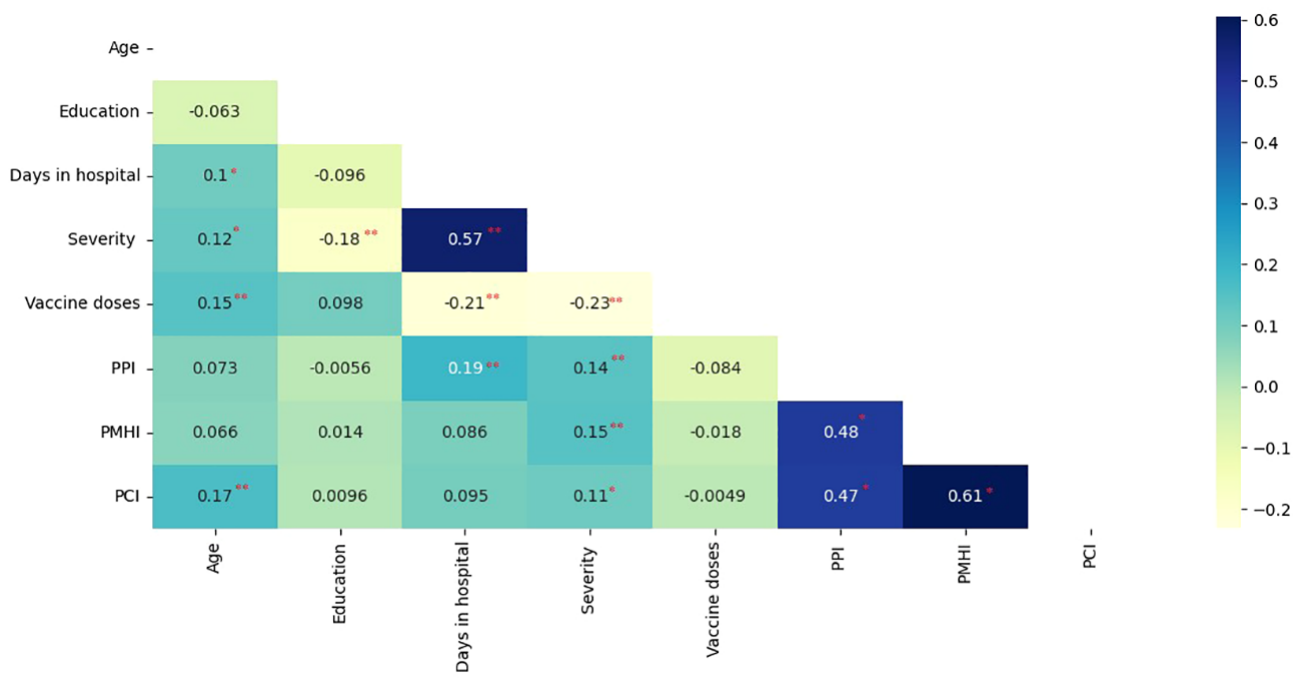
Pearson’s correlation coefficients of perceived health status parameters, clinical, and demographic variables. (PPI, Perceived physical-functioning Impairment; PMHI, Perceived Mental Health Impairment; PCI, Perceived cognitive-functioning Impairment; Correlation is significant at the 0.05 level (2-tailed).*; Correlation is significant at the 0.01 level (2-tailed) **).

Age was positively correlated with perceived decline in physical functioning (p =.001), number of days in hospital (p =.046), severity (p =.017), and number of vaccine doses (p =.005). Education was negatively correlated with severity (p =.001). Duration of hospitalization was positively associated with severity and perceived impairment in physical functioning (p<0.001) and negatively related to the number of vaccine doses (p<.001). Severity was negatively correlated with the number of vaccine doses (p<.001) and positively correlated with perceived decline in physical (p =.006), mental (p =.004), and cognitive functioning (p =.031). The perceived decline in physical functioning was correlated with the perceived decline in mental (p<001) and cognitive functioning (p<001). The latter was found to be strongly associated with perceived impairment in mental health (p<001) (**Figure 1**).

### Results of the in-person interviews and tests

#### Descriptive statistics, risk ratio, and odds ratio

**Figure 2:**
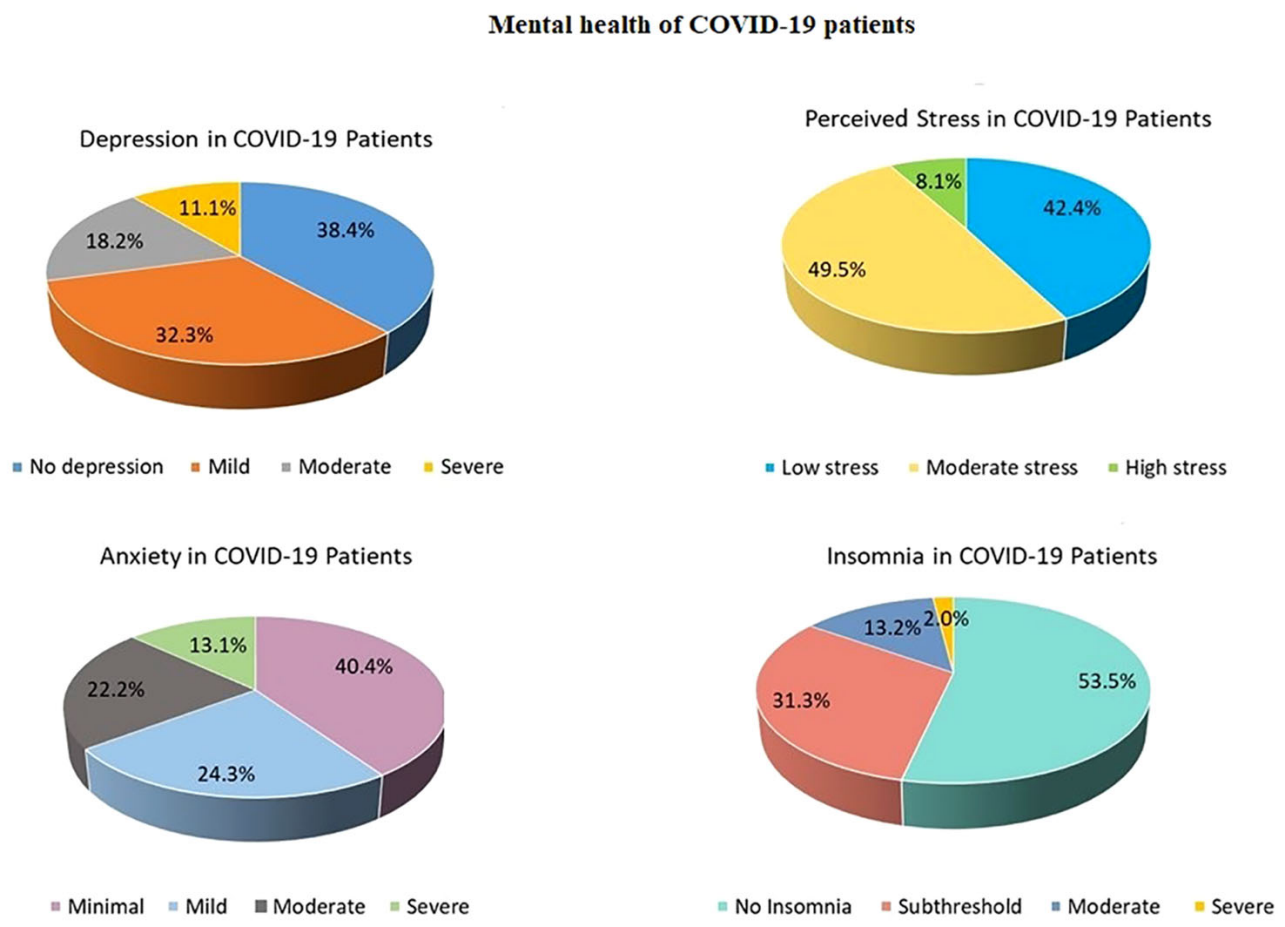
Mental health of COVID-19 patients.

83.8% of the COVID-19 survivors reported at least one of the four symptoms (depression, anxiety, stress, or insomnia) ranging from mild to severe levels (Relative Risk (RR) = 1.37; Odds Ratio (OR) = 3.3). 61% of COVID-19 patients had depression (RR = 1.91; OR = 3.37), 59.6% reported having anxiety (RR = 1.68; OR = 2.68), 57.6% of patients felt stressed (RR = 1.27; OR = 1.65), and 46.5% had sleep problems (RR = 2.4; OR = 3.62) (**Figure 2**). 6.1% of the patients were diagnosed with MCI (RR = 1.94; 95% CI:.242 to 15.5; Z = 0.624; p =.532; OR = 2; 95% CI:.232 to 17.27; Z =.630; p =.528), and 4% were diagnosed with dementia (RR = 2.88; 95% CI:.159 to 52.1; Z =.716; p =.473; OR = 2.97; 95% CI:.156 to 56.68; Z = 0.723; p =.470).

COVID-19 patients with depression and anxiety were more likely to have cognitive decline (<93 on ACE-III) than non-infected controls with similar symptoms. Depression in COVID-19 patients raised the odds of cognitive decline by 8.6 times, and anxiety increased the chances of cognitive decline in COVID-19 patients by 19.4 times. The relative risk of cognitive impairment was also high in COVID-19 patients with depression and anxiety, and the p-values approached significance (**Table 2**).

**Table 2:** Risk ratio and odds ratio.

Mental health problems in COVID-19 survivors					
	Ratio	95% Confidence Interval		Z-value	p-value
Relative Risk		Lower bound	Upper bound		
Overall mental health	1.37	1.02	1.83	2.097	0.036
Depression (BDI)	1.91	1.21	3.26	2.378	0.017
Anxiety (BAI)	1.68	1.02	2.77	2.026	0.043
Stress (PSS)	1.27	0.84	1.94	1.125	0.261
Insomnia (ISI)	2.4	1.14	5.08	2.292	0.022
Odds Ratio					
Overall mental health	3.3	1.33	8.05	2.586	0.01
Depression (BDI)	3.4	1.43	7.93	2.786	0.005
Anxiety (BAI)	2.68	1.16	6.2	2.307	0.021
Stress (PSS)	1.65	0.73	3.71	1.206	0.227
Insomnia (ISI)	3.62	1.36	9.58	2.585	0.01
Co-occurrence of mental health problems and cognitive decline in COVID-19 survivors					
	Ratio	95% Confidence Interval		Z-value	p-value
Relative Risk		Lower bound	Upper bound		
Depression (BDI)	6.8	.97	49.05	1.927	.054
Anxiety (BAI)	15.0	.94	240.7	1.916	.055
Stress (PSS)	5.6	.78	40.5	1.718	.085
Insomnia (ISI)	11.2	.69	181.0	1.702	.088
Odds Ratio					
Depression (BDI)	8.6	1.11	66.45	2.056	.040
Anxiety (BAI)	19.4	1.14	328.5	2,051	,040
Stress (PSS)	6.67	.825	52.14	1.808	.070
Insomnia (ISI)	13.4	.780	228.96	1.789	.073

BDI, Beck’s Depression Inventory; BAI, Beck’s Anxiety Inventory; PSS, Perceived Stress Scale; ISI, Insomnia Severity Index.

It may be noted that over one year had passed between the diagnosis of COVID-19 infection in these patients and our testing (N = 90, M = 463.8 days, SD = 144.9).

#### Comparative analyses of the patient and the control group

**Figure 3:**
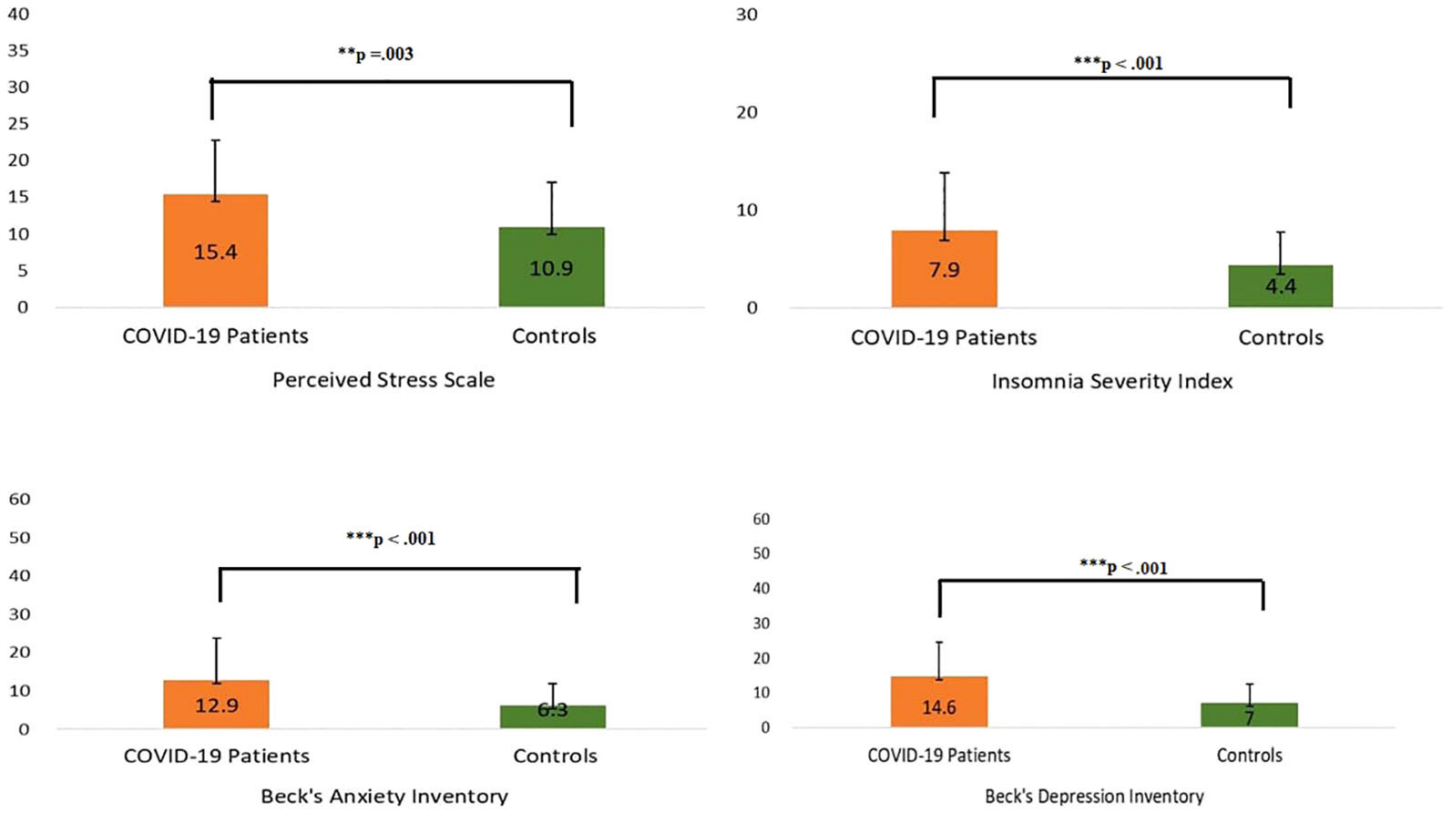
Scores of COVID-19 patients and their matched control participants on mental health screening tools. ** P ≤ 0.01; *** P ≤ 0.001.

The Hotelling’s T^2^-based F-test indicated that there was a statistically significant difference between the two groups (the COVID-19 patients and the non-infected controls) in their mental health status (*T* ^2^ = 18.7, F (4,125) = 4.556, p =.002, partial η2 =.127, observed power =.937). The results of the independent sample t-tests revealed differences between the two groups on the four different mental health parameters (**Table 3**). The patients had higher levels of anxiety (p <.001), depression (p <.001), stress (p =.003), and insomnia (p <.001) compared to the non-infected controls (**Figure 3**).

**Table 3:** Comparative analysis of mental health test scores of COVID-19 patients and control participants.

Mental Health Tests	Group	Mean	SD	t-value	df	p-value	Cohen’s *d*	Effect Size
BDI	COVID-19 Patients	14.6	10.0	5.408	93.47	<.001	.941	Large
	Controls	7.0	5.5					
BAI	COVID-19 Patients	12.9	11.0	4.400	100.3	<.001	.756	Medium
	Controls	6.3	5.6					
PSS	COVID-19 Patients	15.4	7.4	3.031	128	0.003	.663	Medium
	Controls	10.9	6.1					
ISI	COVID-19 Patients	7.9	5.9	4.234	91.855	<.001	.732	Medium
	Controls	4.4	3.3					

BDI, Beck’s Depression Inventory; BAI, Beck’s Anxiety Inventory; PSS, Perceived Stress Scale; ISI, Insomnia Severity Index.

**Figure 4:**
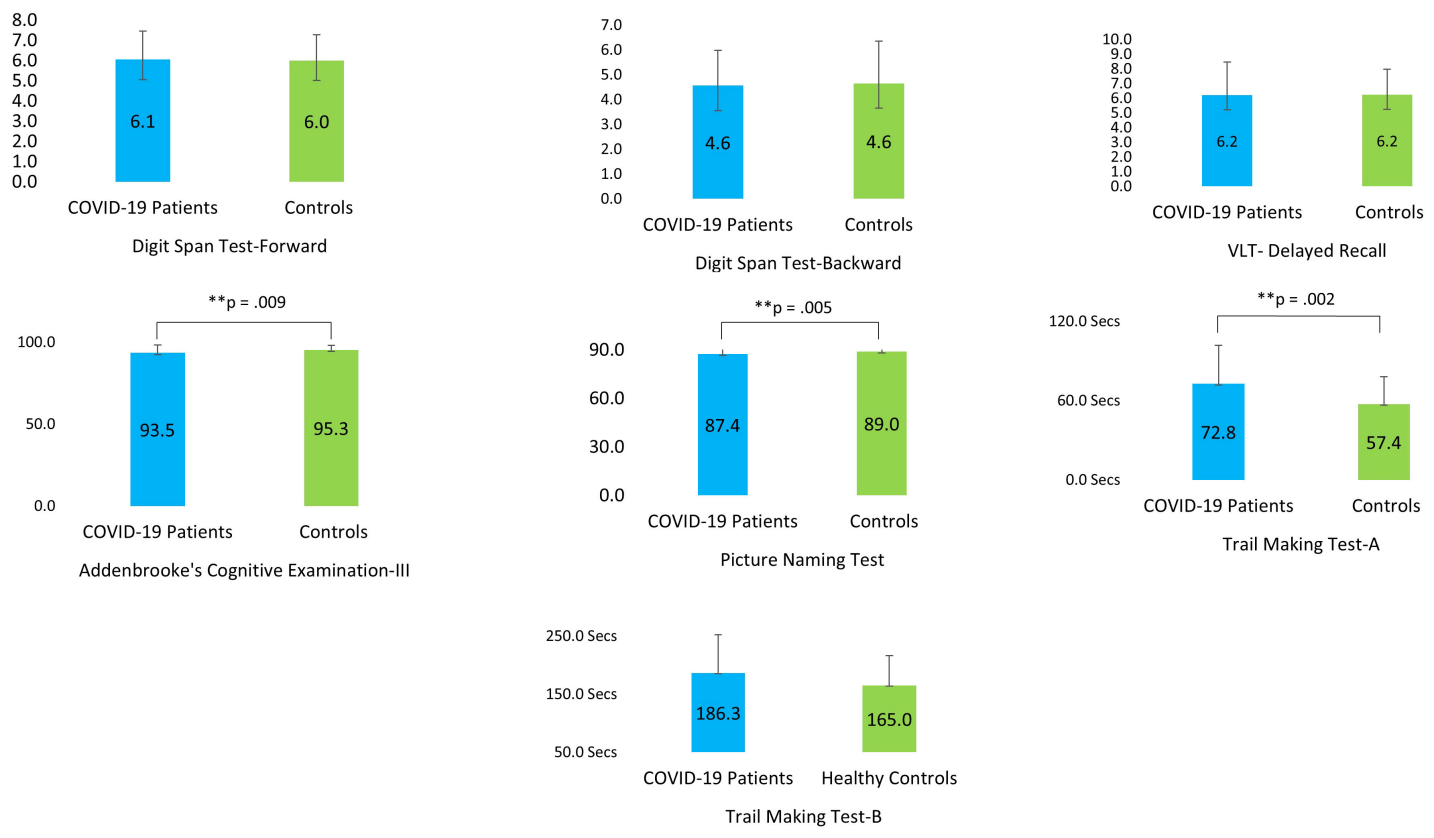
Performance of the COVID-19 patients and their matched control participants on the cognitive tests (VLT, Verbal Learning Test). ** P ≤ 0.01.

Independent sample t-tests indicated that the COVID-19 patients performed significantly worse in ACE-III (p =.009) and PNT (p =.005) compared to the non-infected controls (**Figure 4**). The patients also took significantly longer to complete TMT-A (p =.002) than the control participants (**Table 4**).

**Table 4:** A comparison of cognitive test scores in COVID-19 patients and matched controls.

Test	Group	Mean	SD	t-value	df	p-value	Cohen's d	Effect Size
ACE_III	Patients	93.5	4.8	-2.672	92.7	0.009	0.47	Medium
	Controls	95.3	2.6					
TMT_A	Patients	72.8	29.0	3.281	70.4	0.002	0.61	Medium
	Controls	57.4	20.6					
TMT_B	Patients	186.3	66.0	1.654	128	0.101	0.36	Small
	Controls	165.0	51.0					
Delayed_Recall	Patients	6.2	2.3	-0.077	128	0.939	0	–
	Controls	6.2	1.7					
DST_Forward	Patients	6.1	1.4	0.18	128	0.858	0.07	–
	Controls	6.0	1.3					
DST_Backward	Patients	4.6	1.4	-0.291	128	0.771	0	–
	Controls	4.6	1.7					
PNT	Patients	87.4	4.4	-2.856	116.6	0.005	1.32	Large
	Controls	89.0	1.9					

ACE-III, Addenbrooke’s Cognitive Examination-III; TMT, Trail Making Test; DST, Digit Span Test; PNT, Picture Naming Test.

Among the five different domains that were tested with ACE-III, patients were found to be deficient in language (p =.019) and fluency (p =.003). However, they performed at par with their matched controls in attention (p =.073), memory (p =.361), and visuospatial skills (p =.307) (**Table 5**).

**Table 5:** Performance of COVID -19 patients and matched controls in ACE-III.

	Group	Mean	SD	t-value	df	p-value	Cohen's *d*	Effect-Size
Visuo-spatial	Patients	15.5	1.1	-1.025	130	0.307	0.199	Negligible
	Controls	15.7	0.9					
Fluency	Patients	11.7	1.8	-3.086	83.5	0.003	0.522	Medium
	Controls	12.5	1.2					
Attention	Patients	17.6	0.9	-1.811	90.3	0.073	0.261	Small
	Controls	17.8	0.6					
Memory	Patients	23.0	2.6	-0.916	130	0.361	0.211	Small
	Controls	23.5	2.1					
Language	Patients	25.7	0.5	-2.387	96.7	0.019	0.433	Small
	Controls	25.9	0.3					

#### Linear relationships between different variables: using the Pearson correlation coefficient

COVID-19 severity was significantly correlated with BAI scores. Thus, COVID-19 patients with greater disease severity had higher anxiety levels (r (97) =.211, p =.036). The number of days in the hospital was positively correlated with the processing speed of the TMT-B (r (97) =.200, p =.047) and TMT-A (r (97) =.198, p =.050). Hence, the patients who had to remain under treatment in the hospital for a longer period of time took more time to complete TMT-A and TMT-B. Moreover, patients who had to stay longer in the hospital scored low on DST-forward (r (97) = -.279, p =.005) and PNT (r (97) = -.317, p =.001). BAI was negatively correlated with the scores on ACE-III (r (97) = -.250, p =.013), and BDI was negatively correlated with the scores on forward-DST (r (97) = -.267, p =.008). Therefore, patients who had higher anxiety scored less on ACE-III, and patients who had greater depression scored poorly in the forward DST.

#### Results of the stepwise regression analyses

Exploratory stepwise regression analyses were conducted to investigate if the four demographic variables (age, education, gender, and SES) could predict the four mental health parameters (BDI, BAI, PSS, and ISI). SES was a significant predictor of the level of anxiety (p =.012). Higher socioeconomic status was associated with a lesser amount of anxiety and a better mental health status. However, the effect size was small, and socioeconomic status could only explain 6% of the variance. None of these demographic parameters had any significant effect on BDI, PSS, or ISI (**Table 6**).

**Table 6:** Results of stepwise linear regressions with the demographic variables.

Dependent variable	Predictors	β	t- value	p- value	F-value	df	p-value	R-square	Cohen's(f2)	Effect size
ACE-III	Education	0.313	3.129	0.002	13.495	3, 95	< .001	0.299	0.43	Large
	Age	-0.248	-2.863	0.005						
	SES	0.215	2.163	0.033						
PNT	Education	0.225	2.277	0.025	5.185	1, 97	0.025	0.051	0.05	Small
TMT-A	Age	0.385	4.105	<.001	16.848	1, 97	<.001	0.148	0.17	Medium
TMT -B	Age	0.38	4.246	<.001	15.454	2, 96	<.001	0.244	0.32	Medium
	Education	-0.271	-3.034	0.003						
Delayed Recall	Age	-0.393	-4.433	< .001	16.595	2, 96	<.001	0.257	0.35	Large
	Gender	0.277	3.129	0.002						
DST-Forward	Age	0.247	-2.513	0.014	6.314	1, 97	0.014	0.051	0.05	Small
DST-Backward	Education	0.336	3.669	<.001	12.701	2, 96	<.001	0.201	0.25	Medium
	Age	-0.272	-2.973	0.004						
BAI	SES	-0.25	-2.548	0.012	6.491	1, 97	0.012	0.063	0.07	Small
BDI	None	─	─	─	─	─	─	─	─	NA
PSS	None	─	─	─	─	─	─	─	─	NA
ISI	None	─	─	─	─	─	─	─	─	NA

ACE-III, Addenbrooke’s Cognitive Examination-III; PNT, Picture Naming Test; TMT, Trail Making Test; DST, Digit Span Test; BDI, Beck’s Depression Inventory; BAI, Beck’s Anxiety Inventory; PSS, Perceived Stress Scale; ISI, Insomnia Severity Index.

Stepwise regression analyses were also conducted to predict each of the seven cognitive tests (ACE-III, TMT-A, TMT-B, DST-Forward, DST-Backward, Delayed Recall, and PNT) based on the four demographic variables (**Table 6**). Education, age, and SES were significant predictors of ACE-III scores (p<.001) indicating a higher level of education, a younger age, and a higher socioeconomic status meant better scores on the ACE-III. Higher education was also associated with better performance in PNT (p<.025). However, the size of the effect was small. Age significantly predicted performance on the TMT-A (p<.001). Older age was associated with decreased speed. Consequently, older patients took a longer time to complete TMT-A. Age and education predicted performance on the TMT-B (p<.001). While higher age meant slower processing speed, higher education indicated faster processing. Older age led to worse performance in the delayed recall test, while the female gender was linked to better performance (p<.001). Age significantly predicted performance in the forward digit span task (p =.014). Older age was negatively correlated with performance on this task. Education and age modulated performance on the backward digit span task (p<.001). Age was negatively correlated and education was positively correlated with performance on this task.

## Discussion

While COVID-19 has ushered in a new era of mental health awareness, there has been a paucity of systematic research on the long-term effects of COVID-19 and its mental health consequences. To the best of our knowledge, so far, there has been no empirical study on the Indian population to review and reassess the mental health conditions of COVID-19 survivors. The study addresses this important knowledge gap and tries to understand the mental health outcomes of COVID-19 and its impact on cognitive performance.

A significant number of COVID-19 survivors in our study, especially those with severe infection, perceived a decline in their physical, mental, and cognitive functioning. It must be noted that Li et al. (67) reported a significant association between perceived symptom burden and mental health problems in COVID-19 patients. The number of days in the hospital was associated with a perceived debilitation of physical functioning. Research reports that patients’ perceptions might prove to be more sensitive than the standardized tests that are used at clinics (68). It is important to note that the number of vaccine doses was negatively correlated with severity and the number of days in the hospital. The result is in line with the studies that reported the efficacy and effectiveness of vaccines in reducing infection, severity, hospitalization, and mortality (69).

More detailed in-person interviews and assessments revealed that COVID-19 patients in this study had significant depression, stress, anxiety, and insomnia even when tested over a year after contracting the infection. COVID-19 survivors were 3.3 times more likely to have mental health problems (especially depression, anxiety, and insomnia). This is consistent with prior research that reported mental health problems in COVID-19 patients (6–9, 67). We compared the patients’ scores on the mental health assessments with the control group that was matched for age, gender, education, and socioeconomic background. The patients in our study had greater anxiety, stress, depression, and sleep disorders compared to their matched controls. It might be relevant to note that 60.1 percent and 60.9 percent of the patients reported experiencing a loss of taste and smell, respectively, during the active phase of the infection. Poor olfactory ability has frequently been linked with brain changes in the hippocampus and entorhinal cortex (70–74). Moreover, brain regions involved in processing the hedonic and aversive properties of taste (75), such as the striatum, orbitofrontal cortex, and amygdala, also play a crucial role in emotion processing. Thus, loss of taste and smell in COVID-19 patients could implicate alterations in these brain areas (76) and a consequential disruption of cognitive ability and emotional well-being. It is worth noting that COVID-19 patients with depression and anxiety had 8.6 and 19.4 times greater probabilities of having compromised cognition, respectively, compared to non-infected participants with similar symptoms. This could be due to the differences in the severity of the mental health problems in these two groups.

In the cognitive domain, the COVID-19 patients scored significantly lower compared to their matched controls in ACE-III, PNT, and TMT-A. The ACE-III test measures overall cognition spanning memory, attention, fluency, language, and visual-spatial skills. We ran an exploratory *post hoc* analysis to locate the domains in which the COVID-19 patients underperformed. The patients had significant difficulty in the language and fluency domains. Poor scores in PNT also hint at compromised language abilities. Patients also took much longer to complete TMT-A. TMT-A assesses rote memory, which is linked with recalling factual information or data. Rote rehearsal has been associated with different neural circuits involving the left inferior prefrontal cortex, supplementary motor area (SMA), bilateral posterior parietal cortex, lateral cerebellum, and medial temporal lobe (including the hippocampus) (77). The result seems consistent because letter or semantic fluency involves both strategic and automatic components that recruit the frontal and temporal areas of the brain, respectively (78). Thus, poor scores in TMT-A, language, and fluency might be associated with disruptions in these brain areas.

COVID-19 severity was found to be substantially related to perceived impairment in physical, mental, and cognitive functioning, which is consistent with its association with higher BAI scores. The result is in tune with Rasulo et al., who reported that 50% of ICU survivors had new physical, mental, and/or cognitive problems even one year after their discharge (79). The study is also consistent with a study that stated patients who were bedridden for more than seven days had a higher risk of developing symptoms of depression and anxiety (80). In line with the above studies, we also found that the number of days in the hospital had a significant association with perceived physical impairment. It was also positively correlated with TMT-A and TMT-B, indicating a decline in psychomotor processing speed and cognitive ability, and negatively correlated with the forward DST, signifying a deficit in memory and attention. Thus, patients who stayed longer in the hospital (and were consequently more likely to have greater severity) had a higher probability of having a deficit in short-term verbal memory, focused attention, visual attention, psychomotor processing speed, and executive functioning. Excessive anxiety can impair executive function (81), memory (82), attention (83), and processing speed (84). In concurrence with the above discussion, BAI was negatively correlated with ACE-III, which measures overall cognition.

We investigated how the demographic parameters modulated the health and cognitive status of COVID-19 patients. SES was a significant predictor of anxiety. Patients with higher SES had less anxiety. The result is quite anticipated considering the loss of jobs, dip in incomes, and economic disruptions that the urban poor of this country have endured during and after the pandemic (85, 86). Consequently, patients of low socioeconomic status had to deal with the daily ordeal of eking out a living in these difficult times, apart from coping with the burden of the health problems that COVID-19 has precipitated. The outcome of the cognitive tests raises concern for COVID-19 patients with lower educational levels, higher age groups, and lower socioeconomic status. These three demographic variables significantly predicted the outcome of one of the most commonly used tests for dementia assessment, the ACE-III. Higher age was associated with poor performance in all the cognitive tests except PNT. Similarly, lower education indicated poor performance in the ACE-III, PNT, TMT-B, and DST-Backward tests, which play a crucial role in the diagnosis of dementia. Therefore, COVID-19 patients with lower education and older ages need special attention. Education and age are the two critical factors that modulate the risk of dementia. While lower education has been consistently linked with the risk of dementia (87), older age is reckoned as the biggest risk factor for dementia (88, 89). Low socioeconomic status can also exacerbate the risk of dementia. Marden et al. stated that a stable socioeconomic status in early and adult life “predicted the best memory function and the slowest decline.” (90) The COVID-19 patients in our study had mental health issues as well as cognitive disruptions that might progress to dementia. Close monitoring and regular checkups are needed for early detection of cognitive impairment and timely intervention.

## Limitations

This study has several limitations. We only recruited patients from the databases of three hospitals in Kolkata; thus, our findings have limited generalizability. Furthermore, patients who suffered a decline in physical, mental, and cognitive capacity were more likely to participate in the in-person interview, which may have skewed our findings to some extent. To test cognitive functions, we only included native Bengali speakers with at least ten years of formal education. The exclusion of COVID-19 patients who are uneducated or illiterate is a severe constraint that must be addressed in future studies using more innovative testing procedures designed exclusively for this population. Clinical information other than the severity of the sickness and the number of days in the hospital would have been more valuable; unfortunately, most patients were unable to show any documents other than the hospital’s discharge certificate, and thus we were unable to collect more information. Another significant disadvantage of our study was the unequal size of the groups. During COVID-19 (50), Kolkata was one of the worst-affected districts, accounting for 43.7% of infections together with North 24 Parganas. As a result, obtaining control participants was challenging. Despite these limitations, the study provides crucial and much-needed information about COVID-19 survivors’ mental health and cognitive functioning.

## Conclusion

This study reveals that COVID-19 survivors have significant mental health problems that interfere with their cognitive functioning even one year after contracting the infection. Stringent monitoring and systematic treatment plans should be in place at the earliest possible time to tackle mental problems and related neurocognitive disorders in COVID-19 patients. Efforts must be made to assure that older patients with low education and low socioeconomic status do not fall off the radar of medical surveillance.

## Data Availability

The raw data supporting the conclusions of this article will be made available by the authors, without undue reservation.
